# Draft Genome Sequence of *Thiobacimonas* sp. Strain D13, a Phthalate Ester-Degrading Bacterium Isolated from Deep-Sea Sediments

**DOI:** 10.1128/MRA.00181-19

**Published:** 2019-06-13

**Authors:** Yang Liu, Tuyan Luo, Qiu Lin, Runying Zeng

**Affiliations:** a Institute of Quality Standards and Testing Technology for Agro-Products, Fujian Academy of Agricultural Sciences, Fuzhou, People’s Republic of China; b College of Food and Pharmaceutical Sciences, Ningbo University, Ningbo, People’s Republic of China; c Third Institute of Oceanography, Ministry of Natural Resources, Xiamen, People’s Republic of China; University of Maryland School of Medicine

## Abstract

Thiobacimonas sp. strain D13, newly isolated from the sediments of the southeastern Pacific, can effectively degrade phthalate ester. Here, we report the 5.22-Mbp draft genome sequence of this strain, which will provide insights into the molecular mechanisms underlying its degradation ability.

## ANNOUNCEMENT

Phthalate esters (PAEs) are useful chemical products that are widely applied in the manufacture of pharmaceuticals, household products, etc., as plasticizers. However, PAEs constitute one of the most frequently detected persistent organic pollutants in the environment (1, 2). Known as endocrine-disrupting chemicals, PAEs and their degradation intermediates are reported to pose potential risks to human health even at very low concentrations (3, 4). In this study, we isolated Thiobacimonas sp. strain D13 from the sediments of the southeastern Pacific using artificial seawater (5) supplemented with 600 mg liter^−1^ dimethyl phthalate (DMP) at 30°C. Further study showed that strain D13 could use DMP, diethyl phthalate (DEP), dibutyl phthalates (DBP), or di(2-ethylhexyl) phthalate (DEHP) as the sole carbon source according to the methods described by Ren et al. (6). Chromosomal DNA of strain D13 was isolated and purified using the rapid bacterial genomic DNA isolation kit (Sangon Biotech, Shanghai, China) according to the manufacturer’s instructions. The 16S rRNA gene was amplified with universal primers 27F (5′-AGAGTTTGATCMTGGCTCAG-3′) and 1492R (5′-TACGGYTACCTTGTTACGACTT-3′). Analysis of 16S rRNA gene sequences using blastn (https://blast.ncbi.nlm.nih.gov/Blast.cgi) allowed the assignment of strain D13 to the genus *Thiobacimonas*, with the highest sequence identity (100%) observed with the 16S rRNA gene of Thiobacimonas profunda JLT2016^T^, which was previously designated Salipiger nanhaiensis (7). This genome sequence information for strain D13, known as the second genome sequence available for the genus *Thiobacimonas*, may provide us with fundamental genetic information for understanding the PAE degradation mechanism and its unique physiological characteristics.

The genome of *Thiobacimonas* sp. strain D13 was sequenced using the Illumina HiSeq X Ten sequencing platform at the Shanghai Majorbio Bio-pharm Technology Co., Ltd. (Shanghai, China). At least 2 μg genomic DNA was used in sequencing library construction. Paired-end libraries with an insert size of ∼400 bp were constructed according to the manufacturer’s instructions (Bioo Scientific, AIR paired-end DNA sequencing kit). A total of 1,191-Mbp paired-end reads of 150 bp were generated. All low-quality (Q < 30) data were filtered out using Sickle version 1.33 (8), and the clean reads were subjected to KmerGenie (9) to predict the optimal K value and assembly size. High-quality reads of approximately 797 Mbp, which provided a 152-fold depth of coverage, were assembled with SOAPdenovo version 2.04 (10) using default parameters, resulting in 109 contigs (*N*_50_, 156,822 bp) and rearranged into 69 scaffolds with a total size of 5,217,351 bp. The G+C content of strain D13 was 66.7%.

Protein-coding sequences were predicted by GLIMMER software version 3.02 (http://ccb.jhu.edu/software/glimmer/index.shtml) using default parameters and annotated using BLAST searches of nonredundant protein sequences from the NCBI, Swiss-Prot and TrEMBL (https://www.ebi.ac.uk/uniprot), COG (11), and KEGG (latest version) databases. rRNA genes were detected using Barrnap software version 0.8 (12), and tRNA genes were detected using tRNAscan-SE (13). The draft genome is composed of 5,055 putative protein-coding genes and contains 3 rRNA genes and a total of 46 tRNA genes for all 20 amino acids.

According to the annotation results, a number of genes involved in the degradation of PAEs were identiﬁed in the genome of *Thiobacimonas* sp. strain D13, including genes encoding esterase, protocatechuate 3,4-dioxygenase, 4,5-dihydroxyphthalate dehydrogenase, 4,5-dihydroxyphthalate decarboxylase, and others. In addition, one betaine-aldehyde dehydrogenase gene and one l-ectoine synthase gene, which are important for survival in a saline estuary, were identified. The genome sequence of strain D13 will contribute toward a greater understanding of the molecular mechanisms underlying PAE degradation and provide new perspectives on the ecological functions of *Thiobacimonas* spp.

### Data availability.

The GenBank/EMBL/DDBJ accession number for the 16S rRNA gene sequence of strain D13 is MH997816. This whole-genome shotgun project has been deposited at DDBJ/ENA/GenBank under the accession number RAPJ00000000. The version described in this paper is version RAPJ01000000. Raw sequencing reads are available in NCBI under BioProject accession number PRJNA493210.
