# Correction for Liu et al., “Draft Genome Sequence of *Salipiger* sp. Strain D13, a Phthalate Ester-Degrading Bacterium Isolated from Deep-Sea Sediments”

**DOI:** 10.1128/MRA.00215-20

**Published:** 2020-04-23

**Authors:** Yang Liu, Tuyan Luo, Qiu Lin, Runying Zeng

**Affiliations:** a Institute of Quality Standards and Testing Technology for Agro-Products, Fujian Academy of Agricultural Sciences, Fuzhou, People’s Republic of China; b College of Food and Pharmaceutical Sciences, Ningbo University, Ningbo, People’s Republic of China; c Third Institute of Oceanography, Ministry of Natural Resources, Xiamen, People’s Republic of China

## AUTHOR CORRECTION

Volume 8, no. 24, e00181-19, 2019, https://doi.org/10.1128/MRA.00181-19. The title should read as given in this correction, and *Thiobacimonas* sp. strain D13 should be changed to *Salipiger* sp. strain D13 throughout. At the time the paper was written, strain D13 belonged to the genus *Salipiger*, with 100% similarity to Salipiger nanhaiensis based on the 16S rDNA gene sequences (X. Dai, X. Shi, X. Gao, J. Liang, and X.-H. Zhang, Int J Syst Evol Microbiol 65:1122–1126, 2015, https://doi.org/10.1099/ijs.0.000066). However, according to the modification of Salipiger nanhaiensis by Huang et al. (Z. Huang, Q. Lai, and Z. Shao, Int J Syst Evol Microbiol 67:2043–2045, 2017, https://doi.org/10.1099/ijsem.0.001982), we depicted strain D13 as *Thiobacimonas* sp. Recently, the genus of *Thiobacimonas* was reclassified as genus *Salipiger* (J. S. Wirth and W. B. Whitman, Int J Syst Evol Microbiol 68:2393–2411, 2018, https://doi.org/10.1099/ijsem.0.002833).

